# LIM-Kinases in Synaptic Plasticity, Memory, and Brain Diseases

**DOI:** 10.3390/cells10082079

**Published:** 2021-08-13

**Authors:** Youssif Ben Zablah, Haiwang Zhang, Radu Gugustea, Zhengping Jia

**Affiliations:** 1 Program in Neurosciences and Mental Health, The Hospital for Sick Children, Peter Gilgan Centre for Research and Learning, Toronto, ON M5G 0A4, Canada; youssif.benzablah@sickkids.ca (Y.B.Z.); haiwang.zhang@sickkids.ca (H.Z.); radu.gugustea@sickkids.ca (R.G.); 2 Department of Physiology, Temerty Faculty of Medicine, University of Toronto, Toronto, ON M5S 1A8, Canada

**Keywords:** LIMK, actin, long-term potentiation, long-term depression, memory, brain disorders

## Abstract

Learning and memory require structural and functional modifications of synaptic connections, and synaptic deficits are believed to underlie many brain disorders. The LIM-domain-containing protein kinases (LIMK1 and LIMK2) are key regulators of the actin cytoskeleton by affecting the actin-binding protein, cofilin. In addition, LIMK1 is implicated in the regulation of gene expression by interacting with the cAMP-response element-binding protein. Accumulating evidence indicates that LIMKs are critically involved in brain function and dysfunction. In this paper, we will review studies on the roles and underlying mechanisms of LIMKs in the regulation of long-term potentiation (LTP) and depression (LTD), the most extensively studied forms of long-lasting synaptic plasticity widely regarded as cellular mechanisms underlying learning and memory. We will also discuss the involvement of LIMKs in the regulation of the dendritic spine, the structural basis of synaptic plasticity, and memory formation. Finally, we will discuss recent progress on investigations of LIMKs in neurological and mental disorders, including Alzheimer’s, Parkinson’s, Williams–Beuren syndrome, schizophrenia, and autism spectrum disorders.

## 1. Introduction

Long-term modifications in the efficacy of signal transmission at excitatory synapses, such as long-term potentiation (LTP) and long-term depression (LTD), are considered to be the major cellular mechanisms that contribute to the plasticity of neuronal circuits underlying learning and memory [1,2,3,4,5,6]. One of the key mechanisms for LTP and LTD involves postsynaptic modifications, including changes in size and number of dendritic spines, as well as synaptic trafficking of α-amino-3-hydroxy-5-methyl-4-isoxazolepropionic acid-type glutamate receptors (AMPARs), the principal mediators of excitatory synaptic transmission [7,8,9,10,11,12,13,14,15,16]. In the mammalian central nervous system, most excitatory synapses are located on small dendritic protrusions called dendritic spines, which represent the major postsynaptic component of excitatory synapses [17,18]. Although dendritic spines can be found in various shapes and can alter their morphology during development and synaptic plasticity, mushroom spines with a narrow, short neck and a large distinguishable round head represent the major form of mature spines [19,20]. In addition to mushroom spines, spines may also exhibit other shapes, such as thin spines which lack a clear distinction between the head and neck and stubby spines, with no distinguishable neck. Thin spines represent young, immature spines that are more likely to undergo structural changes [17,18,19]. Dendritic filopodia are protrusions that are believed to actively search for presynaptic partners to initiate neuronal connections during synaptogenesis and are considered precursors of dendritic spines [21,22]. The unique shape of the mature dendritic spine (e.g., large head and narrow neck) is thought to be critical for compartmentalizing local electrical and chemical signals within the spine and restricts diffusion of synaptic molecules out of the spines [20,23,24,25,26,27]. Spine changes, including spine enlargement and shrinkage, are closely associated with LTP, LTD, and memory formation [10,13,20,28,29]. Dendritic spines possess a dense structure called the postsynaptic density (PSD), which is enriched in various synaptic molecules, including AMPARs and associated proteins, allowing for the conversion of synaptic electric signals into biochemical responses to maintain basal synaptic transmission and promote synaptic plasticity [11,17,24,30,31,32,33]. Since the actin cytoskeleton is the major structural component of dendritic spines, many studies have shown that actin reorganization plays a central role in spine formation, maintenance, and dynamic changes under both basal conditions and activity-dependent neural plasticity [31,32,34,35,36,37,38,39,40]. There are two distinct pools of actin filaments within the spine [41]. The stable pool, which is mainly localized at the base of the spine head, is important for the stability of the spine neck, whereas the dynamic pool, localized at the tip of the spine, can generate an expansive force by actin polymerization to mediate activity-dependent enlargement of the spine [28]. As key regulators of the actin cytoskeleton, LIM-domain kinase proteins (LIMKs) play a critical role in synaptic development and plasticity. In addition, studies suggest that LIMK1 could regulate synaptic plasticity and memory by actin-independent mechanisms. Abnormalities in LIMK1 signaling have been reported in multiple neurological and mental disorders. In this review, we will focus on the roles of LIMK1, the most abundant and extensively studied family member, in the regulation of the dendritic spine, synaptic plasticity, memory, and its dysfunction in brain diseases, including Alzheimer’s disease, Parkinson’s disease, Williams–Beuren syndrome, schizophrenia, and autism (Table 1).

## 2. LIM-Domain Kinases (LIMKs) and Their Regulation

LIMKs are a family of serine/threonine protein kinases that are critical for the actin regulation [42,43,44,45]. In addition to the kinase domain at the C-terminus, LIMKs also contain two LIM domains and one PDZ domain at the N-terminus [46,47]. LIM domains can bind to the C-terminal kinase domain and negatively regulate kinase activity [48]. The LIM and PDZ domains also likely affect LIMK function through protein–protein interactions [49]. LIMKs consist of two members, LIMK1 and LIMK2. Both are expressed in multiple tissues in mammals, with LIMK1 being the most abundant in neuronal tissues [42,50]. Studies have shown that LIMK1 is highly expressed within the hippocampus, a brain region critical for learning and memory [51]. Thus, LIMK1 has been the focus of multiple studies in the context of learning and memory. LIMKs are key effectors of the Rho family of small GTPases (e.g., RhoA, Rac1, and Cdc42), the central mediators of actin reorganization in various cell types in response to extracellular and intracellular signals [52,53,54,55,56]. However, it is important to note that LIMKs are not direct substrates of these GTPases, and that activation of Rho proteins does not necessarily lead to the activation of LIMKs. The Rho GTPases regulate LIMKs activity through Rho kinases (ROCKs) and p-21 activated kinases (PAKs) [52,54,57,58]. Both ROCKs and PAKs can directly phosphorylate LIMKs at Thr 508/Thr 505 and increase their kinase activity. In addition to ROCKs and PAKs, protein kinase A (PKA) can also phosphorylate LIMK1 at Ser 323/596 and facilitate its kinase activity [59]. On the other hand, slingshot protein phosphatase (SSH) can directly dephosphorylate LIMKs at Thr 508 and reduce their kinase activity [60]. In addition to kinase activity, the protein level of LIMK1 is also regulated through several mechanisms such as micro-RNA (miRNA)-mediated translation [61] and E3 ubiquitin ligase Rnf 6-dependent proteasomal degradation [62]. Once activated, LIMKs phosphorylate actin-depolymerizing factor, cofilin, at Ser 3 [63,64]. Phosphorylation of cofilin at Ser 3 prevents the binding of cofilin to actin filaments, thus inhibiting filament severing and depolymerization [65,66]. In addition to cofilin, several studies have identified transcription factors as potential targets for LIMK1 in neurons. These include cAMP response element-binding protein (CREB) [67] and Nurr1 [68]. In the case of CREB, which regulates the expression of numerous cyclic-AMP responsive genes by binding to the gene promoter cAMP-response elements, it was shown that the activation of LIMK1 by basic fibroblast growth factor in immortalized hippocampal progenitor cells led to increased CREB phosphorylation and CREB-responsive promoter activity [67]. Nurr1 is an orphan member of the nuclear receptor family that regulates gene transcription via hormone response elements [69]. Purification of Nurr1-binding proteins from immortalized mesencephalic neurons identified LIMK1 as a binding partner, and further analysis revealed that LIMK1 phosphorylated Nurr1 and reduced its transcriptional activity [68]. Therefore, although cofilin is the best characterized LIMK substrate, there are multiple pathways by which LIMKs exert their effects in neurons. This will be further discussed in later sections.

## 3. LIMK1 in Spine Regulation

One of the earliest studies revealing the involvement of LIMK1 in spine regulation comes from Meng and colleagues in 2002, who generated global LIMK1 knockout (KO) mice by homologous recombination. Although these mice showed no changes in the gross anatomy of the CNS, including the hippocampus and cortex, where LIMK1 is highly expressed, LIMK1 KO neurons showed abnormalities in dendritic spines and the actin cytoskeleton [70]. LIMK1 KO hippocampal and cortical neurons had longer, thinner immature spines compared to wild type (e.g., 70–80% of KO spines had a head/neck ratio less than 2, whereas 70–80% of wild type spines had a ratio greater than 2). In addition, the amount of actin filaments in the spine head of LIMK1 KO neurons were reduced and not significantly greater than those of adjacent dendritic areas, as opposed to wild-type neurons where actin filaments were highly enriched in the spines [70]. These data suggest that LIMK1 is critical for the assembly of actin filaments within spines and that abnormalities in the actin cytoskeleton underlie the abnormal spine morphology in LIMK1 KO mice. This conclusion was supported by changes in the activity of cofilin in LIMK1 KO mice, where both basal and activity-dependent cofilin phosphorylation were reduced [70]. Consistent with the genetic study, recent studies using shRNA knockdown showed that LIMK1, specifically LIMK1 palmitoylation at Cys 7/8, plays an important role in actin turnover and spine regulation [71]. In this study, impairment in spine actin turnover was detected immediately following LIMK1 knockdown in hippocampal neurons. In addition, the chronic loss of LIMK1 resulted in spine elimination and reduced spine density by approximately 40%. Reintroduction of wild-type LIMK1, but not a palmitoylation-deficient mutant, rescued both actin turnover and spine density [71]. Although no changes in spine density were detected in LIMK1 KO mice, it is possible that compensatory mechanisms may have occurred in these mice. As discussed earlier, although LIMK1 and LIMK2 have different expression patterns and subcellular localization, they show significant structural and functional similarities, including protein domain organization and their ability to regulate actin dynamics through cofilin [44,72]. Indeed, it has been shown that LIMK1 and LIMK2 double KO mice had more severe deficits in cofilin phosphorylation and synaptic function than LIMK1 and LIMK2 single KO mice, suggesting that LIMK2 may be able to compensate for the loss of LIMK1 [73]. Further studies are needed to investigate this possibility.

In addition to its role in basal spine properties, LIMK1 is also required for the activity-dependent changes in dendritic spines [71]. The knockdown of LIMK1 induced by the focal activation of glutamate receptors using 2-photon (2P) uncaging of glutamate reduced dendritic spine enlargement by 20% [28,71,74]. LIMK1 palmitoylation was also shown to be required for this spine enlargement [71]. How LIMK1 regulates these spine changes during synaptic plasticity remains unknown; however, studies have suggested that the cofilin-dependent actin reorganization may play a key role. It has been shown that following glutamate uncaging, cofilin underwent spine translocation and this process appears to be regulated by cofilin phosphorylation [75,76]. Spine accumulation of cofilin and its subsequent effect on local actin filaments may reduce the density of membrane-proximal actin and triggers new membrane protrusions as shown in other systems [77]. Similarly, the activation of glutamate receptors has been shown to induce accumulation and stabilization of LIMK1 to the juxtamembrane of the dendritic spines through a palmitoylation-dependent mechanism [71].

Other evidence supporting the role of LIMK1 in spine regulation comes from manipulations of its upstream regulators, including PAKs, ROCKs, RhoA, and Rac1. For example, although PAK1 or PAK3 single KO mice showed no structural deficits in spines or synapses [78,79], PAK1/3 double KO mice resulted in longer, thinner spines similar to the ones detected in LIMK1 KO [70,80]. Similarly, studies using antisense and RNA interference to inhibit PAK3 activity revealed abnormalities in dendritic spines, with increased levels of filipodia-like protrusions by approximately 500% and immature spines in rat organotypic slice culture by more than 100% [81]. In addition, PAK2 heterozygous mice had a 30% reduction in spine density that was associated with reduced LIMK1 and cofilin phosphorylation as well as impaired actin polymerization in the cortex and the hippocampus [82]. Cortical neurons in the dominant-negative PAK1 transgenic mice exhibited approximately 20% fewer spines and an increase in the proportion of larger synapses [83] These data suggest that PAKs regulate dendritic spines through LIMK1-dependent mechanisms. Another LIMK1 activator, ROCK2, can also regulate dendritic spines through LIMK1 and cofilin. In one study, using cultured mouse hippocampal slices, neurons treated with the ROCK inhibitors Y-27632 were found to have longer spines (spine length increased by 50%), similar to spines detected in LIMK1 KO neurons [84]. ROCK2 KO mice had reduced synaptic density (by 30%), increased spine length (by 40%), and filipodia-like protrusions, and these spine abnormalities were associated with altered spine actin filaments and reduced cofilin phosphorylation [85].

Numerous studies have shown that the downstream effector of LIMKs, cofilin, is involved in both basal spine properties and spine plasticity [86]. For example, overexpression of a constitutively inactive form of cofilin in hippocampal cultures led to the formation of more mature spines and elevated spine density, whereas overexpression of a constitutively active form of cofilin induced the formation of immature spines [87]. In cofilin conditional KO mice, hippocampal neurons showed a small increase in synapse density (10%) and spine size (20%) [88], and these changes were more pronounced in actin-depolymerization factor (ADF) and cofilin-1 double KO mice [89]. Therefore, the PAK/ROCK-LIMK1-cofilin signaling pathway may represent a key mechanism to regulate basal spine properties and activity-dependent spine plasticity. It is important to note that the results from the manipulations of LIMK1 upstream and downstream regulators only provided indirect evidence that suggests the involvement of LIMK1 in spine regulation. Further studies, such as LIMK1 rescue experiments, are needed to determine whether the effects of these proteins are mediated by LIMK1.

## 4. LIMK1 in Synaptic Plasticity

**Figure 1:**
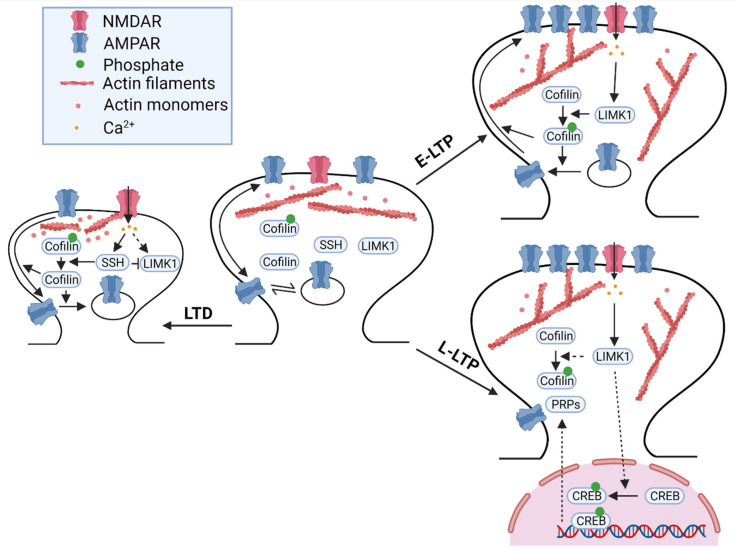
**LIMK1 in synaptic plasticity.** During LTD, the activation of NMDARs and influx of Ca^2+^ activates SSH. Activated SSH dephosphorylates and inactivates LIMK1. In addition, SSH dephosphorylates and activates cofilin, which mediates actin depolymerization. This results in dendritic spine shrinkage, internalization, and lateral movement of AMPARs out of the synapse. During E-LTP, NMDAR activation and Ca^2+^ influx activates LIMK1. Activated LIMK1 phosphorylates and inactivates cofilin, which subsequently leads to actin polymerization. Actin polymerization results in dendritic spine enlargement, insertion, and lateral movement of AMPARs into the synapse. During L-LTP, LIMK1 phosphorylates and activates CREB in the nucleus. The activation of CREB activates the transcription and the translation of plasticity-related proteins (PRPs) which help to maintain synaptic changes, including dendritic spine enlargement and AMPAR distribution.

Numerous studies have shown that LIMKs are involved in both LTP and LTD (Figure 1, Table 1). Electrophysiological recordings in the hippocampal CA1 region showed that although basal synaptic strength was not altered, early-phase long-term potentiation (E-LTP) induced by high-frequency stimulation was enhanced. Importantly, the effect of actin-depolymerizing drugs, such as cytochalasin-D, which also increased E-LTP in wild-type mice, was attenuated in LIMK1 KO mice, suggesting that the role of LIMK1 on E-LTP is mediated by the actin cytoskeleton [70]. No differences in enhanced E-LTP between LIMK1 KO and LIMK1 and LIMK2 double KO mice were observed [73], suggesting that LIMK2 may not play a role in E-LTP. This may be because LIMK1 is more predominantly expressed in the hippocampus and cortex compared to LIMK2 [90]. How LIMK1 regulates E-LTP is unclear, but studies have shown that the LIMK1/cofilin pathway affects the trafficking and accumulation of AMPA receptors at the synapse following LTP induction [88,91].

In addition to its impact on E-LTP, LIMK1 has been shown to regulate late-phase LTP (L-LTP), and this effect appears to be mediated by a cofilin-independent pathway through CREB activation. In 2015, Todorovski and colleagues showed that LIMK1 KO mice exhibited impairments in hippocampal L-LTP. However, the impairment in L-LTP was not mitigated by cofilin peptides designed to increase or decrease cofilin phosphorylation. Interestingly, deficits in L-LTP in LIMK1 KO mice were rescued by increasing CREB activity pharmacologically [92]. Consistent with these data, the L-LTP induction protocol increased CREB phosphorylation, and this activity-dependent CREB activation was attenuated in LIMK1 KO mice. Therefore, LIMK1 appears to play a dual role in LTP regulation: E-LTP through AMPAR trafficking and spine enlargement mediated by cofilin-dependent actin mechanisms and L-LTP through new gene expression and protein synthesis mediated by CREB-dependent mechanisms. How LIMK1 exerts its effects on CREB activation in neurons is yet to be investigated.

The role of LIMK1 in LTP is further supported by studies on its upstream regulators, such as PAK [79] and ROCK [85], and its downstream target cofilin [88,91]. For example, it was shown that both PAK1 KO mice and PAK3 KO mice were impaired in hippocampal LTP [78,79]. Interestingly, L-LTP, but not E-LTP, was impaired in PAK3 KO mice, and this impaired L-LTP was associated with reduced CREB phosphorylation without alteration of cofilin phosphorylation [78]. These results suggest that the PAK3-LIMK1-CREB signaling pathway plays a role in L-LTP regulation. As expected, PAK1 and PAK3 double KO mice had more severe deficits compared to single KO mice with abnormalities in spines and LTP, as well as reduced brain size, neuronal complexity, and altered neuronal excitability [80]. In addition to PAKs, ROCK2 KO mice were also impaired in basal synaptic transmission and LTP that were accompanied by reduced cofilin phosphorylation [85]. Furthermore, in cofilin-1 conditional KO mice, where cofilin-1 is selectively deleted in the excitatory neurons of the postnatal forebrain, decreased LTP was observed in these neurons [88]. Therefore, the PAK/ROCK2-LIMK1-cofilin pathway is clearly important in both hippocampal basal synaptic transmission and plasticity, but whether the effects of PAKs and ROCK2 are mediated by LIMK1 or cofilin remains to be investigated. In addition, how the PAKs/ROCK2-LIMK1-cofilin pathway interacts with the CREB-dependent process to regulate L-LTP requires further investigation.

LIMK1 is also involved in the regulation of LTD through cofilin-dependent mechanisms. Although NMDAR-dependent LTD induced by low-frequency stimulation (LFS) was normal in LIMK1 KO mice [70], metabotropic glutamate receptor-dependent LTD (mGluR-LTD) induced by paired-pulse LFS (PP-LFS) required intact LIMK1-cofilin signaling [93]. mGluR-LTD induced cofilin dephosphorylation and AMPAR internalization, both of which were dependent on the extracellular interaction between GluA2 and cadherin and subsequent activation of Rac1. In GluA2 KO mice, mGluR-LTD was impaired, but this impairment was rescued in LIMK1 KO mice or by manipulations to inhibit cofilin phosphorylation [93]. These results suggest that mGluR activation triggers GluA2-dependent inhibition of Rac1-LIMK1 and dephosphorylation of cofilin, which facilitates AMPAR internalization, spine shrinkage, and mGluR-LTD [93]. In support of this, mGluR-LTD was completely abolished in PAK1 and PAK3 double KO mice [80]. Furthermore, cofilin 1 conditional KO mice showed impairments in LTD [88].

In addition to postsynaptic regulation, LIMK1 is also involved in presynaptic function. LIMK1 KO mice exhibited an enhanced synaptic depression in response to intense neuronal activity and an increased frequency of miniature excitatory postsynaptic currents (mEPSCs), both of which are indicators of increased neurotransmitter release [70]. These results are consistent with the data obtained from ADF and cofilin double KO mice, which exhibited an enhancement in synaptic vesicle exocytosis [89,94]. Furthermore, disruption of Rho proteins, ROCK2, and PAKs also affected synaptic vesicle exocytosis, further supporting the presynaptic role of LIMK1 [79,80,95], but whether the effects of these upstream regulators are mediated by LIMK1 requires more investigation. The presynaptic effects of LIMK1 may be mediated by actin changes as pharmacological perturbations of actin dynamics rescued the increased mEPSCs in LIMK1 KO mice [70]. This possibility is consistent with the results obtained from other studies showing that direct disruption of the actin cytoskeleton affected synaptic vesicle mobilization and exocytosis [34,96].

## 5. Mechanisms Regulating LIMK1 Activity at the Synapse

At many central synapses, the induction of LTP is dependent on postsynaptic Ca^2+^ influx through NMDARs and subsequent activation of Ca^2+^-dependent protein kinases, such as Ca^2+^/calmodulin-dependent protein kinase II (CaMKII), protein kinase C (PKC), and protein kinase A (PKA) [97,98,99]. These signaling molecules ultimately affect the trafficking and channel properties of AMPARs to achieve enhanced synaptic transmission [100,101,102,103]. Similarly, both NMDARs and some of the NMDAR-dependent signaling molecules involved in LTP, such as CaMKII, are required for dendritic spine enlargement [28,104,105]. Induction of LTD and associated spine shrinkage are also dependent on Ca^2+^ influx from NMDARs, although distinct signaling molecules such as protein phosphatases are activated [2,3,106,107]. How is LIMK1 regulated by these NMDAR-dependent signaling processes during synaptic and spine plasticity? Many studies have indicated that LIMK1 is regulated by NMDARs through the Rho GTPase signaling process.

The Rho family of small GTPases (e.g., Rac1, RhoA, and Cdc42) can be activated by NMDARs and CaMKII. For example, NMDAR activation induced activation and translocation of Rac1 to the cell surface in the CA1 region of hippocampal slices [108]. The activity of small GTPases is modulated by guanine-nucleotide-exchange factors (GEFs) that promote the formation of active, GTP-bound state and GTPase-activating proteins (GAPs), which catalyze GTP hydrolysis and the formation of the inactive, GDP-bound state [109]. It was shown that stimulation of NMDARs led to CaMKII-dependent activation of Rac1 via Kalirin, a GEF for Rac1, which was associated with rapid enlargement of dendritic spines, synaptic delivery of AMPARs, and LTP [110]. Other studies showed that Rac1 was activated by NMDARs through Tiam, another Rac1-associated GEF, in a CaMKII-dependent manner [111,112]. Direct manipulations of Rac1 affect spine density and morphology. For example, overexpression of either Rac1 or Rac3 increased spine number [113,114]. Double KO of Rac1 and Rac3 inhibited the formation of dendritic spines and increased filopodia-like spines, similar to the genetic deletion of LIMK1 [114]. Similarly, the activity-dependent activation of RhoA was shown to require NMDARs and Rho-associated GAP p250 [115]. Moreover, RhoB KO mice showed reduced LTP and altered spine morphology, and these changes were accompanied by decreased phosphorylated LIMKs in the hippocampus [116]. These data suggest that LIMK1 is activated by NMDARs through Rho GTPases during spine and synaptic plasticity.

**Figure 2:**
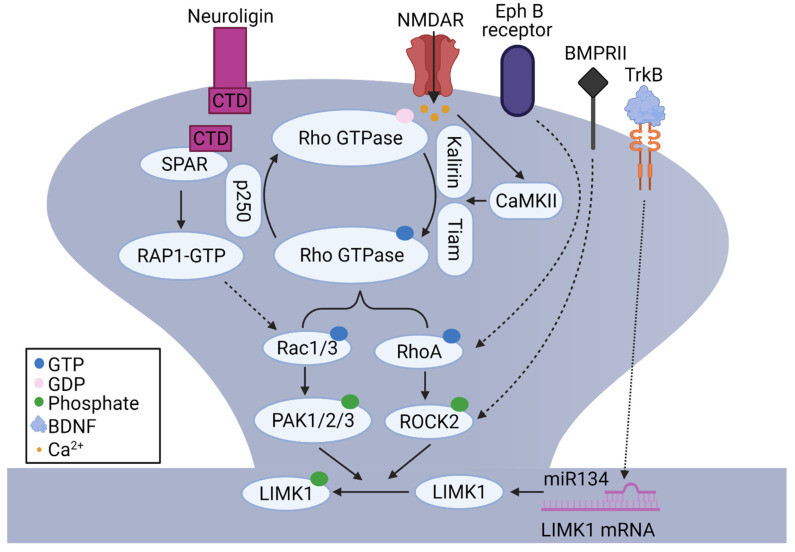
**Signaling pathways regulating LIMK1 activity at the synapse. The activation of NMDARs causes Ca^2+^ influx into dendritic spines.** The increased intracellular Ca^2+^ activates CaMKII, which in turn activates Rac1/3 through Kalirin and Tiam or RhoA through p250. These Rho GTPases bind to and activate PAK1/2/3 and ROCK2, which subsequently phosphorylate and activate LIMK1. LIMK1 can also be activated following NLG1 activation through the SPAR/RAP1/Rac1 pathway. Ephrin receptors can also activate LIMK1 through the RhoA/ROCK2 pathway. In addition, BMPRII receptors can interact with and activate LIMK1. The binding of BDNF to TrkB receptors relieves miR134-mdicated repression of LIMK1 mRNA and induces local translation of LIMK1 within dendrites.

In addition to glutamate receptors, other neuronal surface proteins and receptors have also been shown to regulate the actin cytoskeleton and dendritic spines through LIMK1-dependent mechanisms. For example, neuroligin (NLG1), a postsynaptic cell adhesion molecule, regulates spine density and synaptic plasticity through LIMK1-cofilin mediated actin remodeling [117]. It was shown that neuronal activities induce proteolytic cleavage of NLG1 and the release of its C-terminal domain (CTD), which then interacts with dendritic spine-associated Rap GTPase activating protein (SPAR), activates Rac1, and increases phosphorylated-cofilin. Importantly, the effects of the NLG1 CTD on cofilin phosphorylation, spine enlargement, and synaptic plasticity are eliminated in LIMK1/2 double KO mice, suggesting a central role of LIMK1/2 in these processes [117]. In addition, growth factors and their receptors regulate spine growth and synaptic function through LIMK1-dependent mechanisms. For example, overexpression of neuregulin1 (NRG1), an epidermal growth factor, in cultured hippocampal neurons and in transgenic mice, reduced spine density and frequency of mEPSCs through the activation of LIMK1 and subsequent inactivation of cofilin [118]. Brain-derived neurotrophic factor (BDNF), is another growth factor that induces translational upregulation of LIMK1 in the dendrites, modulates the activity of cofilin, and enhances dendritic spine growth. The effect of BDNF is mediated by relieving the miR134-dependent repression of LIMK1 mRNA translation [61,119]. Another important regulatory pathway to regulate LIMKs involves type II bone morphogenic protein receptor (BMPRII). In rat primary cortical neurons, BMPRII interacts with LIMK1 [120] and affects dendritic growth [121] and influences synaptic stability in *Drosophila* [122]. Other receptors that regulate dendritic spines through the LIMK1 signaling pathway include ephrin receptors. In hippocampal neurons, the activation of EphB receptors affects RhoA activity and cofilin-mediated dendritic spine remodeling [123] and this requires ROCK and LIMK1 [87]. Hormones can also regulate dendritic spines through the LIMK1 signaling pathway. In the hippocampus, estrogen can induce remodeling of the actin cytoskeleton and spines by rapid activation of LIMK1 [124,125,126] and blocking the LIMK1-cofilin pathway eliminates the effects of estrogen on spines and LTP [127,128,129]. In summary, these data suggest that LIMK1 acts as a converging point for multiple signaling pathways to regulate spines and E-LTP through cofilin-dependent actin reorganization (Figure 2). The signaling pathways that regulate LIMK1 and CREB during L-LTP remain largely unexplored, although studies suggest that they may involve PKA and mitogen-activated protein kinase (MAPK), key signaling molecules critical for CREB activation and L-LTP [130]. Both PKA and MAPK have been shown to directly or indirectly affect LIMK1 activity [59,131]. Further studies are required to investigate how these signaling pathways regulate LIMKs.

## 6. LIMK1 in Memory

LTP and LTD are regarded as key mechanisms for learning and memory [1,3,4,6]. The demonstrated role of LIMK1 in these forms of synaptic plasticity suggests that LIMK1 is important in memory, which is supported by several studies. For example, LIMK1 KO mice have enhanced cued fear response in fear conditioning and impaired spatial learning [70]. Similarly, intra-hippocampal administration of a LIMK inhibitor interfered with contextual fear memory acquisition, consolidation, retrieval, and reconsolidation without affecting memory extinction [132]. Furthermore, LIMK1 KO mice were impaired in long-term spatial and fear memory [92], which is consistent with the role of LIMK1 in L-LTP, as discussed earlier. How LIMK1 affects these different forms of memory is unclear, but it may be related to the fact that LIMK1 can regulate multiple synaptic and molecular processes as discussed above. Indeed, pharmacological manipulations of the PKA-CREB signaling pathway, but not of cofilin activity, was able to improve long-term memory performance in LIMK1 KO mice, suggesting that the CREB-dependent mechanism and L-LTP may be particularly important in mediating the effect of LIMK1 in long-term memory [92]. Other mechanisms, including LTD and spine plasticity, as discussed above, may play a role in other forms of memory. Further studies are needed to address these possibilities.

Other indirect evidence supporting the importance of LIMK1 in memory comes from studies on LIMK1 upstream and downstream proteins. Impaired learning and memory have been documented for mice lacking PAK1/2/3, ROCK2, Rho GTPases, and cofilin [80,86,133,134]. For example, PAK1 and PAK3 double KO mice had profound deficits in fear conditioning memory that were associated with LIMK1-cofilin changes [80]. Expression of dominant-negative PAK3 in the entorhinal cortex impaired social recognition memory [135]. Pharmacological inhibition of ROCK2 in the lateral amygdala prior to training significantly impaired long-term memory during fear conditioning [133]. Inhibition of cofilin activity impaired contextual fear memory extinction in rats [86,88,134,136]. It is important to determine whether the memory deficits in these mice are related to LIMK1.

## 7. LIMK1 in Brain Diseases

Given the role of LIMK1 in the regulation of actin dynamics, dendritic spines, synaptic plasticity, and learning and memory, it is not surprising that deficits in LIMK1 are implicated in a wide range of brain disorders [137]. These conditions include Alzheimer’s disease, Parkinson’s disease, Williams–Beuren syndrome, schizophrenia, and autism spectrum disorders. The role of LIMK1 in these diseases will be discussed briefly below.

### 7.1. LIMK1 in Alzheimer’s Disease

Alzheimer’s disease (AD) is an irreversible, progressive neurodegenerative condition characterized by cognitive impairment and memory loss [138,139]. The accumulation of extracellular β-amyloid (Aβ) peptides into senile plaques and neurofibrillary tangles of hyperphosphorylated tau are two pathological hallmarks in AD brains [138,140,141]. In the brain, Aβ results from the proteolytic processing of the amyloid precursor protein (APP) and it has been proposed that the accumulation of toxic Aβ42 plays a major role in the impairment of cognitive functions [141,142]. How Aβ peptides lead to cognitive impairments remains unclear but synapses appear to be a major target underlying dementia [143,144]. Loss of dendritic spines, synapses, and synaptic proteins have been widely reported in animal models of AD [145,146], as well as postmortem brain tissues from AD patients [145,146]. Given the role of the actin cytoskeleton in mediating changes in dendritic spines and synaptic plasticity, multiple studies reported abnormalities in actin networks and underlying regulatory processes [147,148]. For example, a recent study found that Aβ42 peptides induced spine degeneration and neuronal hyperexcitability through LIMK1-dependent mechanisms in rat hippocampal neurons [149]. It was shown that Aβ42 oligomers induced activation of ROCK2 and LIMK1, and that ROCK2 and LIMK1 activity were increased in hAPPJ20 transgenic mice [149,150]. Importantly, pharmacological inhibition of LIMK1 rescued Aβ-induced spine loss and morphological aberrations in the hippocampus [149]. In addition to synaptic effects, it was shown that treatment of rat hippocampal neurons with high levels of fibrillar Aβ (fAβ) induced LIMK1-mediated cofilin phosphorylation, neuritic dystrophy and neuronal cell death, and that inhibition of cofilin phosphorylation prevents neuronal degeneration [151]. Similarly, immunostaining analysis of brain tissues from AD patients showed a significant increase in the number of phosphorylated LIMK1-positive neurons in areas affected with AD pathology [151]. In support of this, several studies also showed elevated levels of inactive phosphorylated cofilin brain tissues from AD patients and mouse models [152,153,154]. Aβ42 oligomers also induced LIMK1 activation through Rac1 and Cdc42 and subsequent activation of PAK1 [155]. Although increased LIMK1 activity is associated with AD, both activation and inactivation of cofilin are observed in AD. For example, Aβ42 oligomers promoted cofilin dephosphorylation in the hippocampus-derived HT22 cell line and primary cortical neurons [156] and reducing cofilin activity rescued Aβ42-induced synaptic protein loss, as well as deficits in LTP and contextual memory in APP/PS1 mice [156]. Aβ42-induced spine loss can be blocked by expression of constitutively inactive cofilin (S3D) [157]. These results suggest that increased LIMK1 activity in AD brains may serve as a compensatory mechanism to reduce cofilin activation caused by other changes, such as cofilin phosphatases SSH. Indeed, it was shown that Aβ42-induced cofilin dephosphorylation in the HT22 cell line was mediated by β1-integrin, a cell receptor important in the maintenance of synapses [158], and the subsequent activation of SSH [156]. Thus, the balanced action of LIMK1 and SSH critical for cofilin regulation may be particularly venerable to Aβ42 effects. In addition to LIMK1, LIMK2 also seems to be involved in AD. Injection of streptozotocin in rats led to inhibition of the neuronal insulin receptor and induced an AD-like phenotype and these effects were associated with increased phosphorylated LIMK2, degeneration of synaptic structures, and memory deficits [159]. Importantly, the effect was abolished by fasudil hydrochloride, a ROCK inhibitor [160], suggesting a role of the ROCK2–LIMK2 pathway. Taken together, these results suggest that dysregulated Rho GTPase–LIMKs-cofilin pathway contributes to the spine, synaptic, and memory deficits of AD and, therefore, targeting this pathway may provide a therapeutic strategy to preserve synaptic function and cognition in AD patients [149,161,162].

### 7.2. LIMK1 in Parkinson’s Disease

Parkinson’s disease (PD) is a progressive neurodegenerative disorder characterized by motor dysfunctions including tremor at rest, rigidity, akinesia (or bradykinesia), and postural instability [163,164]. These pathological symptoms result from the progressive loss of dopaminergic neurons primarily from the substantia nigra pars compacta, the aggregation of α-synuclein in cellular inclusions, and the formation of Lewy bodies in the substantia nigra [165,166]. Studies of the brains of PD patients and animal models have demonstrated that motor deficits are associated with dendritic atrophy, reduced spine density, and abnormal spine morphology in the medium spiny neurons in the striatum [146,167]. In neurotoxin-induced rodent and primate PD models, a marked decrease in the number of spines and alterations in spine head volume in striatal neurons were reported [168,169]. Using the A53T α-synuclein transgenic mouse, a model for early stages of PD showing hyposmia and rapid eye movement sleep behaviour disorders without obvious motor dysfunction, it was demonstrated that thin-type, immature spines were more prevalent compared to WT mice [170]. These data suggest that synaptic impairment occurs during early stages of PD pathology. In addition to the striatum, changes in dendritic spines and synapses were also reported in other brain regions such as the hippocampus [171] and the olfactory bulb [172]. Several studies demonstrated the involvement of LIMK1 in PD. In 2007, Lim and colleagues showed that Parkin, whose mutations are associated with loss of neurons in PD [173], interacts with LIMK1. Parkin ubiquitinates LIMK1 in human dopaminergic neuronal BE(2)-M17 cells but not in HEK cells, suggesting tissue-specific regulation. Furthermore, Parkin reduces LIMK1-mediated cofilin phosphorylation and assembly of actin filaments [174]. Thus, Parkin mutations may contribute to impairments in spines and synaptic function through LIMK1-dependent mechanisms. Leucine-rich repeat serine/threonine-protein kinase (LRRK), another key player in PD [175,176], also interacts with and regulates LIMK1. It was shown that increased kinase activity of LRRK2 reduced neurite outgrowth, whereas LRRK2 deficiency increased neurite length and branching in primary neuronal cultures and in the rat brain [177]. These findings, combined with evidence for high levels of LRRK2 expression in striatal neurons [178] and involvement of LRRK2 in the formation of actin-enriched precursors of dendritic spines during neural development [179], suggest that LRRK2 may contribute to spine deficits through actin regulation. Indeed, it was shown that mutant LRRK2 led to abnormal synaptogenesis and synaptic transmission through LIMK1-cofilin signaling [179]. α-Synuclein is another protein reported to accumulate within Lewy body in PD and shown to regulate LIMK1 signaling. In culture neurons, α-synuclein activated a signaling cascade resulting in cofilin inactivation, stabilization of actin filaments, and axonal and synaptic integrations through the Rac1-LIMK pathway [180]. In addition, increased cell death and reduced survival of newborn neurons in synuclein transgenic animals may be due to an altered actin cytoskeleton and LIMK1 [171]. These studies suggest that LIMK1-cofilin mediated dysregulation of actin dynamics contributes to early deficits in synaptic structure and function that precede neurodegeneration in PD.

### 7.3. LIMK1 in Williams–Beuren Syndrome (WBS)

WBS is a developmental disorder that affects multiple body systems. Genetically, it is caused by a hemizygous deletion of 1.5–1.8 Mb on the chromosome 7q11.23 [181]. Patients affected by WS are characterized by dysmorphic facial features alongside infantile hypercalcemia and abnormalities in connective tissue [182]. Neurologically, individuals with WBS are overly social and have well-developed linguistic skills but severe deficits in learning abilities and visuospatial cognition [182]. Brain anatomy and structure are also affected in WBS. For example, reduction in overall brain and cerebral volume and abnormal distribution of white and grey matter were reported in WBS patients [183,184,185]. Abnormalities in the structure and the function of the amygdala, hippocampus, and cerebral cortex have also been described [183,186,187]. Depression of hippocampal energy metabolism and synaptic activity suggested an abnormal functionality of the hippocampus in WBS [186]. Alterations in brain structure and function have also been reported in different WBS mouse models [188,189,190,191]. For example, mice with the complete deletion (CD) that mimic the most common and recurrent deletion found in WBS patients, showed a significant reduction in brain weight, hippocampus volume, and cellular density of the amygdala [191]. These CD mice presented with many features similar to WBS, such as growth deficiency, craniofacial, and cardiovascular abnormalities, and several behavioural alterations including hypersociability and visuospatial deficits [191]. The fact that these mice have deficits in hippocampal dendritic spines and LTP suggests that these synaptic alterations may underlie the cognitive deficits associated with WBS [191,192].

The neurological phenotypes of WBS could be attributed to the deletion of several genes, including LIMK1, Stx1a, and Clip2 [193]. For example, KO mice of LIMK1, Stx1a, and Clip2 all presented impairments in hippocampal LTP and memory deficits during the contextual fear conditioning test [70,92,194,195]. The fact that only LIMK1 KO mice showed impairments in spatial learning and memory suggests that LIMK1 may be specifically linked to the visuospatial deficits in WBS [70,92,196]. In addition, it was shown that LIMK1 heterozygous mice, which lack only one copy of the LIMK1 gene, as occurred in WBS patients, were also impaired in long-term, but not short-term, memory [92]. These results are consistent with selective impairments in long-term memory associated with WBS, and suggests that LIMK1 is a direct cause of visuospatial memory deficits of this disorder. It remains to be investigated whether LIMK1 KO mice have altered social behaviour as shown in WBS patients. In addition, further experiments are needed to determine whether the synaptic and circuit abnormalities found in LIMK1 KO mice also exist in WBS brains and whether restoration of these abnormalities is able to improve neurological and cognitive deficits in WBS patients.

### 7.4. LIMK1 in Schizophrenia

Actin-based neuronal and synaptic defects including spines and synaptic plasticity are also landmarks of psychiatric disorders, including schizophrenia [197,198,199]. For example, proteins encoded by genes linked to schizophrenia, such as dysbindin, disrupted one in schizophrenia (DISC1) and collapsin response mediator proteins (CRMPs) were found to localize with and regulate the actin cytoskeleton in neurons [200,201,202,203]. In addition, NRG1, whose gene mutations are linked with increased susceptibility to schizophrenia [204,205], affects dendritic spines and synaptic function through interacting and regulating LIMK1 signaling [117,118]. For example, NRG1 transgenic mice, which mimic high levels of NRG1 in excitatory neurons of forebrain in schizophrenic patients, exhibited increased LIMK1 activity and reduced spine density, and pharmacological inhibition of LIMK1 rescued spine density [118]. In patients with schizophrenia, the expression of LIMK1 was dysregulated [206,207], which makes LIMK1 a potential target for therapeutic interventions. For example, through pharmacological inhibition of LIMK1 using Pyr1, it was shown that the synaptic and behavioural deficits, including reduced spine density, impaired LTP, social withdrawal and anxiety-like behaviour in MAP6 KO mice (an animal model of schizophrenia), were rescued [208]. Whether manipulations of LIMK1 signaling pathway have an effect in other animal models of schizophrenia remains to be examined.

### 7.5. LIMK1 in Autism Spectrum Disorders

Autism spectrum disorders (ASD) are complex neurodevelopmental conditions characterized by impairments in social interaction, speech, and non-verbal communication, as well as repetitive and stereotyped behaviours [209,210]. Several genetic syndromes are known to have significant associations with ASD. A prominent example is fragile X syndrome (FXS), the most prevalent single-gene-linked intellectual disability with high incidence of autism [211]. It is caused by mutation in the fragile X mental retardation 1 (*fmr1*) gene on the X chromosome. Fragile X mental retardation protein 1 (FMRP1), the product of the *fmr1* gene, is an RNA-binding protein that plays a key role in the translational regulation of various mRNAs, many of which are involved in the development and maintenance of dendritic spine morphology and synaptic plasticity [212,213,214,215]. Multiple studies suggest that FXS and ASD may share common molecular and cellular mechanisms [215,216,217,218]. In particular, dysregulations of the synaptic actin cytoskeleton, spine morphology, and synaptic plasticity were commonly observed in both ASD and FXS [219,220,221]. Analysis of postmortem brain samples showed a higher spine density in cortical neurons from ASD patients [222]. In addition, disrupted actin regulation at glutamatergic synapses has been reported in various animal models of ASD, and in some cases identified as the underlying cause of ASD-related behavioural phenotypes [223,224,225]. Similarly, several studies have shown that the brain of FXS patients and FMR1 KO mice exhibited abnormal dendritic spine density and morphology [226,227,228]. In line with its function in regulation of the synaptic actin cytoskeleton, considerable evidence suggests that Rac1-PAK-LIMK1 signaling is a point of convergence of several known risk genes linked to ASDs and FXS. Mutations in the Rac1 gene were detected in ASD patients and disruption of Rac1 signaling was shown to contribute to ASD-like behaviours in animal models [224,229,230]. ASD-related aberrant Rac1 activation led to increased spine density [220] and changes in glutamatergic synaptic transmission [230]. Similarly, Rac1 levels/activity were increased in FXS patients [231] and in FMR1 KO mice [232]. In addition, PAKs have been implicated in ASD and FXS [82,233]. Rac1-PAK1-LIMK1-cofilin signaling was increased in the somatosensory cortex of FMR1 KO mice and overexpression of constitutively active cofilin or PAK inhibition rescued aberrant spine morphology and density in the somatosensory cortex of FMR1 KO mice [220]. A recent study suggested that a defect in activity-dependent regulation of cofilin activity and local translation of cofilin mRNA in dendrites may underlie the impairments in structural and functional plasticity in FMR1 KO mice [234]. PAK2 haplo-insufficiency has also been reported in ASD patients and PAK2 heterozygous mice exhibited a marked decrease in synapse density and impaired LTP, as well as autism-relevant behaviours that were related to reduced activity of LIMK1 and subsequent activation of cofilin [82]. In FXS patients, the full-length isoform of bone morphogenetic protein type II receptor (BMPR2) was abnormally high and heterozygosity for BMPR2 or pharmacological inhibition of LIMK1 reduced the density of immature spines and restored synaptic function in FMR1 KO mice [235,236]. Taken together, these studies suggest that LIMK1 may serve as a converging point of aberrant processes associated with ASD and FXS and, therefore, could be used as a therapeutic target.

## 8. Concluding Remarks

In summary, accumulating evidence indicates that LIMK1/2 are a common effector of many signaling pathways in the brain. Through their effects on cofilin and transcription factors, LIMK1/2 regulate actin reorganization, spine properties, synaptic plasticity, and memory formation (Figure 1 and Figure 2, Table 1). In addition, changes in LIMK signaling, including upstream regulators and downstream targets, are widely reported in brain disorders and in some cases, manipulations of LIMK1 improve synaptic and behavioural functions associated with these disorders. However, despite this progress, several issues remain to be addressed. (1) Is the subcellular distribution of LIMK1 regulated by synaptic activity? Although it has been shown that cofilin is dynamically regulated within spines during LTP and LTD, whether and how LIMK1 is also translocated to the spines during these processes remains to be determined. (2) How LIMK1 regulates CREB to control L-LTP and memory? It would be important to determine whether LIMK1 is transported to the nucleus and if so, how this process is regulated during synaptic plasticity and how it is related to cofilin. It is interesting to note that LIMK1 contains a nuclear localization motif within its kinase domain and its nuclear translocation has been reported in other cell types [237,238]. (3) How LIMK1-mediated actin dynamics are associated with and affect behaviour in living animals? In this respect, photoactivatable cofilin and Rac1 may be used to rapidly stimulate the LIMK-cofilin signaling pathway, although Rac1 activation is not specific to this pathway. (4) What is the translational potential to treat brain disorders by manipulating LIMK1? To facilitate this, detailed analysis of LIMK1 changes in various brain regions and cell types is necessary. It is expected that a better understanding of LIMK signaling pathways will not only provide important insight into synaptic structure and function, but also the treatment of related brain diseases.

## Figures and Tables

**Table 1:** Summary of key studies on LIMK1.

Experimental Model and Procedure	Spine Properties	Synaptic Function	Behaviour, Learning, and Memory	Mechanism
Meng et al., 2002 LIMK1 KO mice; cultured neurons; slices	-Reduced mature spines -Increased immature spines	-Enhanced mEPSC frequency and LTP	-Increased locomotor activity -Enhanced cued fear response	-Impaired basal and activity-induced change of p-cofilin -Abnormal actin
George et al., 2015 Rats; cultured neurons; LIMK1 knockdown	-Impaired spine density and plasticity -Rescued by LIMK1 overexpression	N/A	N/A	-Palmitoylation and spine translocation of LIMK1
Meng et al., 2004 LIMK2 KO; LIMK1 and LIMK2 DK mice; slices	N/A	-Enhanced basal synaptic transmission and LTP in LIMK1/LIMK2 DK mice	N/A	-Impaired p-cofilin in LIMK1/LIMK2 DK mice
Meng et al., 2005 PAK3 KO mice; cultured neurons; slices	Normal dendritic/spine morphology	-Normal E-LTP -Impaired L-LTP	N/A	-Normal basal level of p-cofilin -Impaired basal level of pCREB
Asrar et al., 2009 PAK1 KO mice; cultured neurons; slices	-Normal synaptic/spine structures	-Normal LTD -Impaired LTP	N/A	-Impaired activity-induced change of p-cofilin -Abnormal actin
Boda et al., 2004 Mice; cultured slices; PAK3 knockdown	-Increased immature spines	N/A	N/A	N/A
Wang et al., 2018 PAK2 KO mice; slices	-Reduced spine and synapse density	N/A	N/A	-Impaired basal levels of p-cofilin and pLIMK1
Hayashi et al., 2004 Transgenic DN PAK; slices	-Reduced spine density and increased spine size	N/A	N/A	N/A
Tashiro et al., 2004 Mice; cultured slices; inhibition of ROCK	-Reduced spine density -Increased spine length	N/A	N/A	N/A
Zhou et al., 2009 ROCK2 KO mice; slices	-Reduced synaptic density -Increased spine length	-Impaired basal synaptic transmission -Impaired LTP -Normal LTD	N/A	-Impaired basal level of p-cofilin -Normal activity-induced change of p-cofilin
Shi et al., 2009 Mice; cultured neurons; cofilin S3A and S3D expression	-Increased immature spines by cofilin S3A	N/A	N/A	N/A
Rust et al., 2010 Conditional n-cofilin KO mice; slices	-Increased spine density, length, and width	-Impaired L-LTP -LTD resistance	-Impaired spatial and fear learning and memory	N/A
Wolf et al., 2015 Conditional ADF/cofilin DK mice; slices	-Reduced synapse/spine density -Increased spine size	-Impaired PPF -Faster synaptic depression	N/A	-Increased F/G-actin ratio -Impaired synaptic actin dynamics
Todorovski et al., 2015 LIMK1 KO mice; slices	N/A	-Impaired L-LTP, rescued by PKA activator	-Impaired spatial and contextual fear LTM -Rescued by PKA activator	-Normal basal pCREB -Impaired activity-induced change in pCREB
Huang et al., 2011 PAK1/PAK3 DK mice; slices	-Reduced spine density -Increased spine size	-Enhanced basal synaptic transmission -Impaired LTP -Impaired LTD	-Increased locomotor activity and anxiety -Impaired spatial and fear memory	N/A
Lunardi et al., 2018 Rats; LIMK1 inhibitor	N/A	N/A	-Impaired contextual fear memory	N/A
Wang et al., 2013 Rats; cofilin peptides (S3 and pS3)	N/A	N/A	-Cofilin S3 and pS3 enhanced and impaired memory extinction, respectively	N/A
Pennucci et al., 2019 Rac1/Rac3 and Rac3 DK mice; cultured neurons	-Reduced dendritic spines -Increased filipodia	N/A	N/A	N/A
McNair et al., 2010 RhoB KO mice	-Reduced spine density -Increased spine size	-Reduced E-LTP -Normal L-LTP	N/A	-Impaired level of pLIMK1
Henderson et al., 2019 hAPP mice; cultured neurons; Aβ42 treatment	-Reduced spine density -Rescued by LIMK1 inhibitor	-Increased excitability -Rescued by LIMK1 inhibitor	N/A	-ROCK2-LIMK1-dependent mechanism
Heredia et al., 2006 Mice cultured neurons; human tissue	-Neuronal degeneration -Rescued by LIMK1 inhibitor	N/A	N/A	-Increased pLIMK1 level
Woo et al., 2015 Transgenic APP/PS1 mice; cultured neurons	-Synapse loss -Rescued by cofilin inhibitor	-Impaired LTP -Rescued by cofilin inhibitor	-Impaired fear memory -Rescued by cofilin inhibitor	-Increased cofilin dephosphorylation by Aβ42 oligomers
Hou et al., 2012 STZ-model Rats	-Synapse loss -Rescued by ROCK inhibitor	N/A	-Impaired learning and memory -Rescued by ROCK inhibitor	-Increased level of p-LIMK2 and p-cofilin
Segura-Puimedon et al., 2014 WBS-mice	-Reduced spine density	-Impaired LTP	-Enhanced sociability and visuospatial deficits	N/A
Hoogenraad et al., 2002 Clip2 KO mice	N/A	-Impaired LTP	-Impaired contextual fear memory	N/A
Fujiwara et al., 2006 HPC-1/syntaxin 1A KO mice	N/A	-Impaired LTP	-Impaired fear LTM	N/A
Gory-Fauré et al., 2021 MAP6 KO mice	-Reduced spine density -Rescued by LIMK1 inhibitor	-Impaired LTP -Rescued by LIMK1 inhibitor	N/A	N/A
Pyronneau et al., 2017 Fmr1 KO mice	-Increased immature spine density -Rescued by PAK inhibitor	-Reduced mEPSC frequency -Rescued by PAK inhibitor	-Impaired sensory processing -Rescued by PAK inhibitor	-Increased Rac1-PAK1-LIMK1-cofilin signaling

## Data Availability

Not applicable.
